# Hazardous Compounds in Tobacco Smoke

**DOI:** 10.3390/ijerph8020613

**Published:** 2011-02-23

**Authors:** Reinskje Talhout, Thomas Schulz, Ewa Florek, Jan van Benthem, Piet Wester, Antoon Opperhuizen

**Affiliations:** 1 Laboratory for Health Protection Research, National Institute for Public Health and the Environment (RIVM), P.O. Box 1, 3720 BA Bilthoven, The Netherlands; E-Mails: Jan.van.Benthem@rivm.nl (J.v.B.); Piet.Wester@rivm.nl (P.W.); Antoon.Opperhuizen@rivm.nl (A.O.); 2 Bundesinstitut für Risikobewertung, Thielallee 88-92, 14195 Berlin, Germany; E-Mail: Thomas.Schulz@bfr.bund.de; 3 Laboratory of Environmental Research, Department of Toxicology, University of Medical Sciences, Dojazd 30, 60-631 Poznan, Poland; E-Mail: eflorek@ump.edu.pl; 4 Centre for Substances and Integrated Risk Assessment, National Institute for Public Health and the Environment (RIVM), P.O. Box 1, 3720 BA Bilthoven, The Netherlands

**Keywords:** smoke component, risk assessment, tobacco product regulation, Hoffmann list, TTC

## Abstract

Tobacco smoke is a toxic and carcinogenic mixture of more than 5,000 chemicals. The present article provides a list of 98 hazardous smoke components, based on an extensive literature search for known smoke components and their human health inhalation risks. An electronic database of smoke components containing more than 2,200 entries was generated. Emission levels in mainstream smoke have been found for 542 of the components and a human inhalation risk value for 98 components. As components with potential carcinogenic, cardiovascular and respiratory effects have been included, the three major smoke-related causes of death are all covered by the list. Given that the currently used Hoffmann list of hazardous smoke components is based on data from the 1990s and only includes carcinogens, it is recommended that the current list of 98 hazardous components is used for regulatory purposes instead. To enable risk assessment of components not covered by this list, thresholds of toxicological concern (TTC) have been established from the inhalation risk values found: 0.0018 μg day^−1^ for all risks, and 1.2 μg day^−1^ for all risks excluding carcinogenicity, the latter being similar to previously reported inhalation TTCs.

## 1. Introduction

Tobacco smoke is a complex, dynamic and reactive mixture containing an estimated 5,000 chemicals [1–3]. This toxic and carcinogenic mixture is probably the most significant source of toxic chemical exposure and chemically mediated disease in humans [4,5]. According to WHO estimates, 5.4 million premature deaths are attributable to tobacco smoking worldwide [6]. If current trends continue, 10 million smokers per year are anticipated to die by 2025 [7,8]. The most common tobacco smoke related causes of death are cardiovascular disease, chronic obstructive pulmonary disease, and various types of cancer, in particular lung cancer [9]. In addition, environmental tobacco smoke also significantly increases the risk to develop these and other diseases [10]. Obviously, there is a need for regulation of this addictive and harmful product as are most other addictive and/or hazardous products to which the population is exposed. Nevertheless, as yet tobacco products are only loosely regulated and largely exempt from any safety standards.

The WHO Framework Convention on Tobacco Control (FCTC) provides a comprehensive framework for global tobacco control efforts. The FCTC covers all aspects of tobacco control, including tobacco product regulation, advertising, health warnings, price and tax issues, illicit trade (smuggling) and programs for smoking cessation. Article 9 of FCTC addresses the regulation of the contents of tobacco products, including their emissions. The implementation of article 9 requires product regulation measures based on the empirical testing of tobacco products using standardized methods. It is not feasible to measure all 5,000 cigarette smoke components for product monitoring and subsequent regulation purposes. Therefore, a list of smoke components needs to be selected with a sufficiently broad chemical, toxicological, and pharmacological profile.

Currently, both the tobacco industry and authorities strongly focus on the so-called Hoffmann analytes. Hoffmann and his co-workers have published several lists with varying numbers of biologically or toxicologically active mainstream smoke components, which are colloquially referred to as Hoffmann analytes [1,11,12]. The list of Hoffmann analytes is, however, not state-of-the art, as it is based on research from the early 1990s. Furthermore, the Hoffmann publications give no arguments for inclusion of the listed components apart from general statements that these components are biologically active components in mainstream smoke, or that they are carcinogens or major tobacco smoke components. Finally, no endpoints other than carcinogenicity are specified, whereas cancer is only one of three major tobacco-related diseases. Other toxicological endpoints such as those related to cardiovascular and pulmonary disease need to be included as well.

For these reasons we propose that the Hoffmann list needed to be revised. The present paper describes the development of an up-to-date list of hazardous tobacco smoke components together with inhalation risk values covering all major tobacco-related diseases. Many literature data are available on the presence of chemical components in cigarette smoke, often with concentration ranges and occasionally with information on the toxic potency of these components. However, to the best of our knowledge, an exhaustive list of smoke components was not available at the start of the project. Therefore, a database has been generated by reviewing recent literature on smoke components. From our database components with known potential health risks for cancer or other endpoints (primarily cardiovascular and respiratory effects) have been selected as an initial list for regulatory purposes.

## 2. Experimental Section

### 2.1. Database Composition

To screen for smoke components, peer-reviewed literature dating back to 1990 has been searched using PubMed and Scopus. The following search query was used: (“cigarette smoke” OR “tobacco smoke” OR “mainstream smoke”) AND (toxin OR analyte* OR constituent* OR deliveries OR composition* OR component* OR compound* OR “gas phase” OR particulate OR toxin* OR “smoke chemistry” OR emission*).

In addition, all issues of *Recent Advances in Tobacco Science* have been checked, as well as all issues of the journal *Beitraege zur Tabakforschung International* dating back to 1990. Existing lists such as the Hoffmann list, the WHO TobReg list [13], the Rodgman & Green list [14], and the Fowles and Dybing list [15] have also been used. Finally, several textbooks on smoke composition have been consulted [10,16–22].

Information retrieved from these data sources were entered in an Excel database. The database contains detailed information on each chemical compound and its levels in mainstream tobacco smoke, if available. Available human inhalation risk values (cancer and non-cancer risk, safety factors included) from the International Toxicity Estimates for Risk Assessment (ITER) database have also been incorporated. ITER is an Internet database of chronic human health risk values and cancer classifications for over 542 chemicals of environmental concern obtained from several independent organizations worldwide (http://www.tera.org/ITER). This database is updated on a regular basis and contains risk values from the Agency for Toxic Substances and Disease Registry of the Centers for Disease Control of the United States (ATSDR), Health Canada (HC), International Agency for Research on Cancer (IARC), NSF International, Rijksinstituut voor Volksgezondheid en Milieu (RIVM; National Institute of Public Health and the Environment, the Netherlands), and the United States Environmental Protection Agency (U.S. EPA). When more than one of these institutes has published a risk value, the lowest value was selected for our database. In addition, risk values from the Californian Environmental Protection Agency (Cal EPA) as listed in the articles of Fowles and Dybing [23] and Rodgman and Green [14], were included. One NATA (U.S. EPA National-scale Air Toxics Assessment) and two ORNL (U.S. EPA Department of Energy, Office of Environmental Management) values listed in Rodgman and Green were also included.

### 2.2. Derivation of Smoke Components Threshold of Toxicological Concern

From the available inhalation risk values for tobacco smoke components thresholds of toxicological concern (TTC) have been established. Two TTCs were derived, one from all risk values including carcinogens, and one for endpoints other than carcinogenicity. To derive the threshold of toxicological concern, the 5th percentile benchmark dose was taken from the plot of the cumulative probability versus the inhalation risk values. It should be noted that risk values from different agencies were used, which may affect the accuracy of our TTC. Agencies often base their risk assessment on different toxicological data, and apply different safety factors.

## 3. Results and Discussion

### 3.1. Database and List of Hazardous Smoke Components

Our literature search resulted in a database of 2,256 different smoke components. For 542 of these components, yields per cigarette in mainstream smoke were also reported in literature. For the other compounds, only the presence in smoke was mentioned, but the amount not specified. To assess the human health risk of a specific smoke component, data on its smoke yield and inhalation risk value are required. For 98 components, risk assessment authorities have established a human inhalation risk value for cancer and/or another endpoint: 60 cancer and 48 non-cancer inhalation risk values have been found. These 98 components were selected for our list of hazardous smoke components, as their potential hazard contribution can be assessed. Table 1 lists these components, together with their inhalation risk values and the institute that published this value. Searching the recent publication on tobacco and tobacco smoke components by Rodgman and Perfetti [24], containing references to around 5,300 smoke components, may result in hazardous smoke components not yet on our list. Emission levels are known from literature for all 98 components except for five that had been measured but not quantified in smoke. Exposure to the components on this list forms a potential health risk to develop cancer and/or other diseases, primarily cardiovascular and respiratory illnesses.

Our list of hazardous smoke components includes all nine components reported in mainstream cigarette smoke that are known human carcinogens (IARC Group I carcinogens), as well as all nine components that are probably carcinogenic to humans (IARC Group 2A carcinogens) [25,26]. In addition, it contains 34 of the 48 components that are possibly carcinogenic to humans (IARC Group 2B carcinogens) [27].

The WHO Study Group on Tobacco Product Regulation (TobReg) recently published an expert advice on smoke component regulation (based on research by a joint WHO and IARC working group) [13,28]. A list of 43 priority toxicants was composed from three smoke component emission level datasets which were all based on the Hoffmann list. All components of this TobReg initial group of priority toxicants are present on our list, with the exception of catechol, crotonaldehyde, hydroquinone, and NNK. Those components are not on the current list as no human inhalation risk values were found. Catechol has been classified by IARC as possibly carcinogenic to humans (Group 2B); hydroquinone and crotonaldehyde have been classified by IARC as not classifiable as to its carcinogenicity to humans (Group 3).

Considering this classification, these components probably do not form the highest carcinogenic risk of all components in tobacco smoke. Only NNK, that has been classified as Group 1 since 2007 (before 2B) would be worthwhile to include after determining a risk value. In the TobReg article, no non-cancer hazard indices are mentioned. Thus, our shortlist of 98 potentially hazardous smoke components includes all important smoke components from these previous lists. Compared to the Hoffmann list, our list includes many new components including acetone, acetonitrile, cadmium, methyl chloride, methyl ethyl ketone, propionaldehyde and toluene.

### 3.2. Threshold of Toxicological Concern in Smoke Risk Assessment

As human health inhalation risk values have been found for only 98 of the 2,256 smoke components, the potential hazard contribution can only be assessed for these components when using classical risk assessment criteria. An alternative approach is to look at smoke components with an emission level below the threshold of toxicological concern (TTC). The TTC refers to a human exposure threshold below which there would be no appreciable risk to human health, despite the absence of chemical-specific toxicity data [29,30]. When a chemical would be present at concentrations below this level, it can be exempted from further hazard consideration. The TTC is usually a cut-off value based on a set of experimental data, e.g., the 5th percentile value of the distribution of a set of no-observed effect levels (NOEL). TTCs can be defined for several endpoints, the most sensitive being mutagenicity.

The inhalation exposure-based TTC for tobacco smoke components was established at 0.0009 μg m^−3^ for all risk values including those for carcinogens (5th percentile benchmark dose), and 0.06 μg m^−3^ for risk values excluding carcinogenic components. These concentrations can be remodeled to daily doses of respectively 0.0018 and 1.2 μg day^−1^ by assuming a default breathing rate of 20 m^3^ day^−1^. It should be noted that the compounds for which we found human inhalation risk values have been assessed because they are known or suspected toxicants (selection bias). This means that, had our entire dataset been tested for toxicity, the TTC would have turned out higher. Below, our TTC for non-carcinogenic effects is compared to inhalation exposure based TTCs found in literature.

Escher *et al*. report an inhalation TTC for non-carcinogenic endpoints of 4–180 μg day^−1^ (depending on the Cramer class of the component) based on repeated dose toxicity studies from the REPDOSE database [31,32]. No observed effect concentrations (NOECs) have been normalized to daily exposure, and converted to daily doses using a default breathing rate of 20 m^3^ day^−1^ and a safety factor of 25; organophosphates and compounds with a genotoxic structural alerts were excluded. Their value is comparable in magnitude to our TTC for non-carcinogenic components.

Carthew *et al.* derived a TTC for inhalation exposure to aerosol ingredients in consumer products from an inhalation toxicology database of over 100 rodent studies [33]. Using a safety factor of 25, they derived a TTC of 300 μg day^−1^ for systemic effects and a TTC of 1,000 μg day^−1^ for local effects. Genotoxic carcinogens and *in vivo* mutagens have been excluded from their analysis, as well as heavy metals, dioxins, polychlorinated biphenyls, organophosphates and polymers. This may explain why their TTC values are higher (250 and 830 times) than our TTC for non-carcinogenic components.

Based on analysis of toxicological data for hundreds of carcinogenic and noncarcinogenic substances, the FDA derived a human TTC for oral exposure of 1.5 μg day^−1^. Drew and Frangos remodeled this value to an air guideline TTC of 0.03 μg m^−3^ assuming default breathing factors, and 100% absorption in the lungs [34]. Next, they compared this inhalation TTC to air guideline values established by reputable authorities. Their air guideline database was comprised of organics only and did not include carcinogens, sensory irritants, metals, particulates, and dioxins. For the chronic air guideline values established by risk assessment authorities, there are three guideline values lower than the TTC and 280 at or above the TTC. For 3,274 acute air guideline values established by various authorities and from occupational exposure limits, only one value was below the TTC. Thus, the FDA human TTC for oral exposure, 1.5 μg day^−1^, seems to result in a reasonable estimation for inhalatory exposure to non-carcinogens, as well. Our TTC for non-carcinogens (1.2 μg day^−1^) is almost equal to the FDA value.

Thus, the non-carcinogens TTC we derived from inhalation risk values for smoke components is comparable to previously reported inhalation TTCs for non-carcinogenic effects. For 542 components in our database, a concentration range in smoke is known. As a smoker consumes on average 20 cigarettes per day, these levels have to be multiplied by 20 to estimate a smoker’s daily exposure. For 81 of these components, the concentration in smoke is lower than the TTC for all endpoints including carcinogenicity. If the TTC approach would also be valid for the complex mixture of tobacco smoke, this means that for 15% (81/542) of the components with known concentration in smoke, there would be no appreciable risk for any disease including cancer. As a first approximation, these components could be exempted from further hazard consideration, especially if one considers that as many as 461 (542 – 81) smoke components with known concentration levels are present at levels above the TTC and would therefore have a higher priority for hazard characterization anyway. However, one has to take into account that the TTC approach has been developed for exposure to single components or simple mixtures. The complex tobacco smoke mixture, on the other hand, contains more than 5,000 components. Any effects of these components could be antagonistic, independent, additive, or even synergistic, depending on the specific mechanisms of toxicity. Price *et al.* modeled an independent and an additive approach for some simple model mixtures [35]. Further research could study this problem for the much more complex tobacco smoke mixture.

For 172 of these components, the concentration in smoke is lower than the TTC for endpoints other than carcinogenicity. Thus, for 32% (172/542) of the components with known concentration in smoke, there would be no appreciable risk at diseases other than cancer. These components could be exempted from further hazard consideration if they are proven non-carcinogens and/or have no structural alerts for carcinogenicity.

In conclusion, we have derived two inhalation TTCs, one for all risks, including carcinogenicity, and one for endpoints other than carcinogenicity, the latter being well comparable to previously reported inhalation TTCs for non-carcinogenic effects. Only a small part of the smoke components with known yields have emission levels below these TTCs.

### 3.3. How to Use the Initial List for Tobacco Product Regulation?

Our list of 98 smoke components provides a scientific basis for the progressive reduction in the level of toxic chemicals in tobacco emissions. The WHO TobReg expert advice on smoke component regulation proposed lowering of toxicants levels per mg nicotine in cigarette smoke [13,28]. First, the levels for selected smoke components would need to be established and second, sale or import of cigarette brands that have yields above these levels could be prohibited. The Centre for Disease Control in Atlanta already implemented a similar approach by monitoring the levels of three categories of chemicals (tobacco-specific nitrosamines, polycyclic aromatic hydrocarbons, and volatile organic compounds) in tobacco smoke and setting a target to reduce the unit-based sales-weighted average levels of each category by 10% [36].

As the risk for tobacco smoke-related diseases appears to be dose-dependent, reducing the concentration of the most important toxicants in smoke may lower the risks related to tobacco smoking [37]. This harm reduction approach is interesting because in many countries smoking prevalence seems to stabilize after an initial steep decline secured by various policy measures. However, the effect on mortality and morbidity of lowering (classes of) toxins in cigarette smoke has not been clarified yet, one of the reasons being the relatively long lag time for developing tobacco-related diseases. Additional studies are required to assess individual- or population-level reductions in exposure or in adverse health effects. For instance, the consumption of modified cigarettes cannot be linked easily to a reduction in disease risk or even to significant reductions in carcinogen exposure biomarkers [37]. Second, past experiences with the introduction of low-yield tobacco products showed unforeseen effects that counteracted any harm reduction effects. The resulting products did not lead to reduced exposure as consumers adapted their smoking behavior such as frequency and intensity of use to inhale sufficient nicotine to satisfy their craving and addiction [38,39]. On the other hand, consumers did perceive these products to be less hazardous due to marketing health claims, such as ‘light’ and ‘mild’ [40–42]. As these circumstances may lead to a negative health impact, TobReg also advised to report toxicant levels normalized for nicotine level and to prevent marketing of products with reduced toxicant levels as such. Such normalization may lead to less focus on the quantity of smoke generated per cigarette, and on TNCO values as misleading measures of human exposure and risk. On the other hand, toxicant emission levels for cigarettes with different nicotine emission levels can be better compared. According to TobReg, normalization may shift the interpretation of the measurement towards product characterization of smoke toxicity generated under standardized conditions.

In addition to the potential health effect of a smoke component, other criteria are important in selecting components for regulation (e.g., [13,28]). First, the component must have substantial variability in its yield across brands on the market to allow for banning of part of the products. Second and somewhat related, the variation across brands should be substantially greater than the variation in repeat measurement for the component for a single brand. Otherwise, large numbers of measurements would be required for each component in order to tighten the estimation of the mean value, and the cost of testing would increase proportionally. Third, compounds from different chemical classes need to be included. Analyses of variation in brands of 13 mainstream smoke emissions suggests the occurrence of risk swapping (in which one specific exposure is reduced within a group at the cost of another’s exposure increasing) and risk shifting (in which a specific exposure is reduced within a group at the cost of that exposure’s increasing within another group) [43]. For instance, when polycyclic aromatic hydrocarbons are reduced by enhancing nitrate content in tobacco, more tobacco specific nitrosamines are generated in smoke. Therefore, it is warranted that marker components of all relevant chemical classes are included on a list for regulatory purposes.

A final consideration to select smoke components for regulation is the availability of technology, or other approaches, that can reduce the level of specific smoke components, as setting limits on these toxicants then becomes feasible and therefore of higher priority. Some smoke component emission levels may be lowered by adapting agricultural practices, plant characteristics, tobacco blending, and cigarette design (for example additives, filters, papers) [44]. For instance, parameters which influence heavy metal concentration in tobacco include growing conditions (e.g., soil type and pH), agricultural practices (e.g., use of metal-containing pesticides and fertilizers), genotype, stalk position, and processing of tobacco leaves [45]. Another example is the formation of carbonyls in tobacco smoke by the pyrolysis of tobacco components, including celluloses and sugars. Sugar levels in tobacco can be reduced by using different curing methods, and regulating the amount that is added in the manufacturing process [46]. A third example is the yield of many organics in smoke that can be influenced by the type of filter, e.g., charcoal filters remove up to 70% of benzene from cigarette smoke [11].

The current shortlist is solely based on toxicity data from publicly available databases. Thus, other toxic smoke components may be present in our database, but do not appear on the shortlist due to lack of an inhalation risk value. Apart from that, additives and their resulting smoke components may also increase tobacco-related harm by making cigarettes more palatable, attractive, or even addictive to consumers. From a regulatory point of view, identifying smoke components that influence addictiveness of tobacco products is also essential. In addition, smoke components that increase the attractiveness of a tobacco product by affecting e.g., taste, smell and other sensory attributes also need to be cautiously regulated as these may entice more individuals to start or to continue smoking.

Some of the components in Table 1 or in our database are not only toxic, but also increase the addictiveness or the attractiveness of a cigarette. For instance, aldehydes such as acetaldehyde may play a role in cigarette addiction as do the components harman and norharman present in our database [47]. Other components may affect the taste of tobacco smoke to a high extent and thus its attractiveness. One example is 5-hydroxymethylfurfural, a characteristic taste component of Maillard reactions [48]. Unfortunately no sufficient evidence is available on smoke components’ addictiveness or attractiveness or on appropriate methods to acquire these data [49]. Therefore, future research should also focus on these two aspects of tobacco smoking.

In conclusion, our initial list of 98 smoke components can be used for regulatory purposes like the progressive reduction in the level of toxic chemicals in tobacco emissions. A further selection from these 98 components can be made based on criteria such as the variability of the toxicants across brands, the potential for the toxicant to be lowered, the need to include components from different chemical classes, and any attractiveness- or addictiveness-enhancing effects of components.

## 4. Conclusions

Here we provide a list of 98 hazardous smoke components (Table 1) which is based on an extensive literature search for known smoke components and their human health inhalatory risk. This list provides a scientific basis for the progressive reduction in the level of toxic chemicals in tobacco product emissions, through periodic setting of standards. It is advised to replace the Hoffmann list by the current list of hazardous smoke components. As components with potential cardiovascular and respiratory effects have also been included, the three major smoke-related causes of death are all covered by the list. Future updating of this list can be carried out as needed. Based on the inhalatory risks, we also derived two thresholds of toxicological concern (TTCs), one for all risks including carcinogenicity, and one for endpoints other than carcinogenicity, which is well comparable to previously reported inhalation TTCs for non-carcinogenic effects. Only a small part of the smoke components with known yields has emission levels below these TTCs.

Many components on our list (e.g., styrene, acetamide, and methyl chloride) have not been systematically studied in benchmark experiments comprising a variety of brands available on the market, and should therefore be monitored. When these data have been generated, the variability of the toxicants across brands, and the potential for the toxicant to be lowered, can be evaluated. It is therefore recommended that the list of hazardous smoke components be monitored in several brands using different smoking regimes. For many components validated methods are already available from e.g., International Organisation for Standardization (ISO) or Health Canada. For other components, such methods need to be developed or modified from other applications. In the framework of FCTC, harmonized and validated standards will be developed for measuring NNK, NNN, acetaldehyde, acrolein, benzene, benzo(a)pyrene, 1,3-butadiene, carbon monoxide, and formaldehyde.

Once the list of components has been further studied and monitored, and the results have been evaluated, a further selection from the shortlist can be made for regulatory purposes. Here, other criteria such as the variability of the toxicants across brands, the potential for the toxicant to be lowered, the need to include components from different chemical classes, and any attractiveness- or addictiveness-enhancing effects of components can be incorporated. Routine collection and analysis of selected smoke components will accelerate advancement in tobacco control.

## Figures and Tables

**Table 1.** List of hazardous tobacco smoke components with their cancer and non-cancer inhalation risk values.

**Smoke component**	**Cancer risk value^1^(mg m^−3^)**	**Institute**	**Non-cancer risk value^2^(mg m^−3^)**	**Endpoint**	**Institute**
1,1,1-Trichloro-2,2-bis(4-chlorophenyl)ethane (DDT)	1.0E-04	U.S. EPA			
1,1-Dimethylhydrazine	2.0E-06	ORNL			
1,3-Butadiene	3E-04	U.S. EPA	2E-03	reproduction	U.S. EPA
2,3,7,8-Tetrachlorodibenzo-*p*-dioxin (TEQ)	2.6E-04	Cal EPA			
2-Amino-3-methyl-9*H*-pyrido[2,3-*b*]indole (MeAaC)	2.9E-05	Cal EPA			
2-Amino-3-methylimidazo[4,5-b]quinoline (IQ)	2.5E-05	Cal EPA			
2-Amino-6-methyl[1,2-a:3′,2″-d]imidazole (GLu-P-1)	7.1E-06	Cal EPA			
2-Aminodipyrido[1,2-a:3′,2″-d]imidazole (GLu-P-2)	2.5E-05	Cal EPA			
2-Aminonaphthalene	2.0E-05	Cal EPA			
2-Nitropropane		Cal EPA	0.02	liver, focal vacuolization and nodules	U.S. EPA
2-Toluidine	2.0E-04	Cal EPA			
3-Amino-1,4-dimethyl-5H-pyrido [4,3-b]indole (Trp-P-1)	1.4E-06	Cal EPA			
3-Amino-1-methyl-5H-pyrido[4,3-b]-indole (Trp-P-2)	1.1E-05	Cal EPA			
4-Aminobiphenyl	1.7E-06	Cal EPA			
5-Methylchrysene	9.1E-06	Cal EPA			
7*H*-Dibenzo(c,g)carbazole	9.1E-06	Cal EPA			
2-Amino-9*H*-pyrido[2,3-b]indole (AaC)	8.8E-05	Cal EPA			
Acetaldehyde	4.5E-03	U.S. EPA	9.0E-03	nasal olfactory epithelial lesions	U.S. EPA
Acetamide	5.0E-04	Cal EPA			
Acetone			30	neurological effects	ATSDR
Acetonitrile			0.06	mortality	U.S. EPA
Acrolein			2.0E-05	nasal lesions	U.S. EPA
Acrylamide	8E-3				
Acrylic acid			1.0E-03	nasal olfactory epithelium degeneration	U.S. EPA
Acrylonitrile	1.5E-04	U.S. EPA	2.0E-03	respiratory effects	U.S. EPA
Ammonia			0.1	respiratory effects	U.S. EPA
Aniline	B2—probable human carcinogen	U.S. EPA	1E-3	immune-related	U.S. EPA
Arsenic	2.3E-06	U.S. EPA			
Benz[a]anthracene	9.1E-05	Cal EPA			
Benzene	1.3E-03	U.S. EPA	9.8E-03	decreased lymphocyte count	ATSDR
Benzo[a]pyrene	9.1E-06	Cal EPA			
Benzo[j]fluoranthene	9.1E-05	Cal EPA			
Beryllium	4.2E-06				
Cadmium	5.6E-06	U.S. EPA			
Carbazole	1.8E-03	NATA			
Carbon disulfide			0.1	effects on CNS	HC
Carbon monoxide			10	cardiotoxic	Cal EPA
Chloroform,	4.3E-04	U.S. EPA	0.1	liver changes	ATSDR
Chromium VI	8.3E-07	U.S. EPA	1.0E-04	lower respiratory effects	U.S. EPA
Chrysene	9.1E-04	Cal EPA			
Cobalt			5.0E-04	respiratory functions	RIVM
Copper			1.0E-03	lung and immune system effects	RIVM
Di(2-ethylhexyl) phthalate	4.2E-03	Cal EPA			
Dibenzo[a,i]pyrene	9.1E-07	Cal EPA			
Dibenzo[a,h]acridine	9.1E-05	Cal EPA			
Dibenzo[*a,h*]anthracene	8.3E-06	Cal EPA			
Dibenzo[a,j]acridine	9.1E-05	Cal EPA			
Dibenzo[a,h]pyrene	9.1E-07	Cal EPA			
Dibenzo[a,l)pyrene	9.1E-07	Cal EPA			
Dibenzo[a,e]pyrene	9.1E-06	Cal EPA			
Dibenzo[c,g]carbazole	9.1E-06	Cal EPA			
Dimethylformamide			3.0E-02	digestive disturbances; minimal hepatic changes	U.S. EPA
Ethyl carbamate	3.5E-05	Cal EPA			
Ethylbenzene			0.77	liver and kidney effects	RIVM
Ethylene oxide	1.1E-04	Cal EPA			
Ethylenethiourea	7.7E-04	Cal EPA			
Formaldehyde	7.7E-04	U.S. EPA	1.0E-02	nasal irritation	ATSDR
Hexane			0.7	neurotoxicity	U.S. EPA
Hydrazine	2.0E-06	U.S. EPA	5E-3	fatty liver changes	ATSDR
Hydrogen cyanide			3.0E-03	CNS and thyroid effects	U.S. EPA
Hydrogen sulfide			2E-3	nasal lesions	U.S. EPA
Indeno(1,2,3-c,d)pyrene	9.1E-05	Cal EPA			
Isopropylbenzene			0.4	increased kidney, adrenal gland weights	U.S. EPA
Lead	8.3E-04	Cal EPA	1.5E-3	not applicable	U.S. EPA
Manganese			5.0E-05	neurobehavioral	U.S. EPA
*m*-Cresol			0.17	CNS	RIVM
Mercury			2.0E-04	nervous system	U.S. EPA
Methyl chloride			0.09	cerebellar lesions	U.S. EPA
Methyl ethyl ketone			5	developmental toxicity	U.S. EPA
Naphtalene			3E-3	nasal effects	U.S. EPA
*N*-nitrosodi-*n*-butylamine (NBUA)	6.3E-06	U.S. EPA			
*N*-nitrosodimethylamine (NDMA)	7.1E-07	U.S. EPA			
Nickel			9.0E-05	chronic active inflammation and lung fibrosis	ATSDR
Nitrogen dioxide			1.0E-01	not applicable	U.S. EPA
*N*-nitrosodiethanolamine	1.3E-05	Cal EPA			
*N*-nitrosodiethylamine	2.3E-07	U.S. EPA			
*N*-nitrosoethylmethylamine	1.6E-06	Cal EPA			
*N*-Nitrosonornicotine (NNN)	2.5E-05	Cal EPA			
*N*-Nitroso-*N*-propylamine	5.0E-06	Cal EPA			
*N*-nitrosopiperidine	3.7E-06	Cal EPA			
*N*-nitrosopyrrolidine	1.6E-05	U.S. EPA			
*n*-Propylbenzene			0.4	increased organ weight	U.S. EPA
*o*-Cresol	C- possible human carcinogen	U.S. EPA	0.17	decreased body weight, neurotoxicity	RIVM
*p*-, *m*-Xylene			0.1	respiratory, neurological, developmental	U.S. EPA
*p*-Benzoquinone	C- possible human carcinogen	U.S. EPA	0.17	CNS	RIVM
*p*-Cresol	C- possible human carcinogen	U.S. EPA	0.17	CNS	RIVM
Phenol			0.02	liver enzymes, lungs, kidneys, and cardiovascular system	RIVM
Polonium-210	925.9	ORNL^3^			
Propionaldehyde			8.0E-03	atrophy of olfactory epithelium	U.S. EPA
Propylene oxide	2.7E-03	U.S. EPA			
Pyridine			0.12	odour threshold	RIVM
Selenium			8E-4	respiratory effects	Cal EPA
Styrene			0.092	body weight changes and neurotoxic effects	HC
Toluene			0.3	colour vision impairment	ATSDR
Trichloroethylene	82	HC	0.2	liver, kidney, CNS effects	RIVM
Triethylamine			7.0E-03	n.a.	U.S. EPA
Vinyl acetate			0.2	nasal lesions	U.S. EPA
Vinyl chloride	1.1E-03	U.S. EPA			

1 Cancer inhalation risk values provide an excess lifetime exposure risk, in this case the human lung cancer risk at a 1 in 100,000 (E-5) level.

2 Noncancer inhalation risk values indicate levels and exposure times at which no adverse effect is expected; here values for continuous lifetime exposure are listed.

3 Unit risk in risk/pCi = 1.08E-08.
